# High Fidelity of Mouse Models Mimicking Human Genetic Skeletal Disorders

**DOI:** 10.3389/fendo.2019.00934

**Published:** 2020-02-04

**Authors:** Robert Brommage, Claes Ohlsson

**Affiliations:** ^1^Department of Internal Medicine and Clinical Nutrition, Centre for Bone and Arthritis Research, Institute of Medicine, The Sahlgrenska Academy at University of Gothenburg, Gothenburg, Sweden; ^2^Department of Drug Treatment, Sahlgrenska University Hospital, Gothenburg, Sweden

**Keywords:** skeletal dysplasia, skeletome, mouse models, genetic disease, nosology

## Abstract

The 2019 International Skeletal Dysplasia Society nosology update lists 441 genes for which mutations result in rare human skeletal disorders. These genes code for enzymes (33%), scaffolding proteins (18%), signal transduction proteins (16%), transcription factors (14%), cilia proteins (8%), extracellular matrix proteins (5%), and membrane transporters (4%). Skeletal disorders include aggrecanopathies, channelopathies, ciliopathies, cohesinopathies, laminopathies, linkeropathies, lysosomal storage diseases, protein-folding and RNA splicing defects, and ribosomopathies. With the goal of evaluating the ability of mouse models to mimic these human genetic skeletal disorders, a PubMed literature search identified 260 genes for which mutant mice were examined for skeletal phenotypes. These mouse models included spontaneous and ENU-induced mutants, global and conditional gene knockouts, and transgenic mice with gene over-expression or specific base-pair substitutions. The human X-linked gene *ARSE* and small nuclear RNA *U4ATAC*, a component of the minor spliceosome, do not have mouse homologs. Mouse skeletal phenotypes mimicking human skeletal disorders were observed in 249 of the 260 genes (96%) for which comparisons are possible. A supplemental table in spreadsheet format provides PubMed weblinks to representative publications of mutant mouse skeletal phenotypes. Mutations in 11 mouse genes (*Ccn6, Cyp2r1, Flna, Galns, Gna13, Lemd3, Manba, Mnx1, Nsd1, Plod1, Smarcal1*) do not result in similar skeletal phenotypes observed with mutations of the homologous human genes. These discrepancies can result from failure of mouse models to mimic the exact human gene mutations. There are no obvious commonalities among these 11 genes. Body BMD and/or radiologic dysmorphology phenotypes were successfully identified for 28 genes by the International Mouse Phenotyping Consortium (IMPC). Forward genetics using ENU mouse mutagenesis successfully identified 37 nosology gene phenotypes. Since many human genetic disorders involve hypomorphic, gain-of-function, dominant-negative and intronic mutations, future studies will undoubtedly utilize CRISPR/Cas9 technology to examine transgenic mice having genes modified to exactly mimic variant human sequences. Mutant mice will increasingly be employed for drug development studies designed to treat human genetic skeletal disorders.

**Significance:**

Great progress is being made identifying mutant genes responsible for human rare genetic skeletal disorders and mouse models for genes affecting bone mass, architecture, mineralization and strength. This review organizes data for 441 human genetic bone disorders with regard to heredity, gene function, molecular pathways, and fidelity of relevant mouse models to mimic the human skeletal disorders. PubMed weblinks to citations of 249 successful mouse models are provided.

## Introduction

Rare human genetic diseases cumulatively affect about 1 in 200 individuals and involve an estimated 7,000 genes. Major research efforts are underway to identify these mutant genes and characterize their disease phenotypes. Knowledge gained can guide therapies and provide hypotheses to develop future treatments. As recently summarized (1), “Genome sequencing has revolutionized the diagnosis of genetic diseases. Close collaborations between basic scientists and clinical genomicists are now needed to link genetic variants with disease causation. To facilitate such collaborations, we recommend prioritizing clinically relevant genes for functional studies, developing reference variant-phenotype databases, adopting phenotype description standards, and promoting data sharing.”

**Figure 1:**
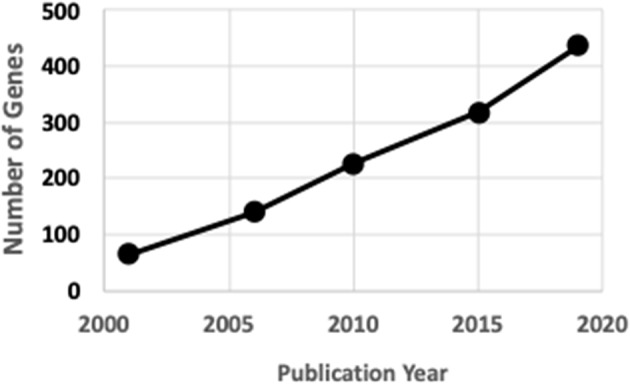
ISDS Nosology gene identification.

Rare human genetic skeletal dysplasias affect about 1 in 5,000 individuals (2) and account for 5% of all birth defects (3). The International Skeletal Dysplasia Society (ISDS, https://www.isds.ch), promotes scientific progress in the field of skeletal dysplasias and dysostoses, meets every second year, and published skeletal nosology summaries during 2001 (4), 2006 (5), 2010 (6), 2015 (7), and 2019 (8). There are presently 441 skeletal nosology genes, with an average of 20 new genes identified yearly (Figure 1). The classification aims to (i) identify metabolic pathways active in cartilage and bone, and their regulatory mechanisms; (ii) identify cellular signaling networks and gene expression sequences implicated in skeletal development; (iii) identify candidate genes for genetic disorders; (iv) facilitate integration of data coming from spontaneous and genetically engineered mouse mutants; (v) help in developing diagnostic strategies; (vi) stimulate the design and exploration of new therapeutic possibilities; and (vii) provide a knowledge framework accessible to physicians as well as to basic scientists and thus to facilitate communication between clinical genetics and pediatrics and the basic sciences (4).

The objectives of the present review include further characterizations of these 441 skeletal nosology genes and evaluating the reliability of mutant mouse models to mimic these human skeletal disorders.

## Historical Highlights

Short stature and other visually obvious skeletal dysplasias were apparent throughout human history (9). The discovery of X-rays by Wilhelm Röntgen (10) was quickly followed by the description of osteopetrosis by Albers-Schönberg (11) and many skeletal dysplasias during the following decades (12). Dual-energy X-ray absorptiometry (DXA) technology, developed during the 1980s (13), permitting quantitation of bone mineral density (BMD), and continued advances in computed tomography (CT), providing 3 dimensional images, lead to increasing sophisticated understanding of bone dysmorphology. The first nosology gene identified was *CA2* (carbonic anhydrase 2, osteopetrosis), initially in 1983 using electrophoretic, enzymatic and immunologic techniques on red blood cell extracts (14), and subsequently by genetic mutation analysis in 1991 (15). The first genetic mutation for any human disease to be identified by WES was *DHODH* (dihydroorotate dehydrogenase), responsible for postaxial acrofacial dysostosis, in 2010 (16).

## Nosology

Nosology is the classification of diseases, which in its simplest form involves symptoms and pathogenic mechanisms. No classification system is perfect and there are often multiple ways to classify a given disorder. At the extremes, “lumpers” and “splitters” prefer few and many categories, respectively (17). Heredity can be X-linked, autosomal dominant, or autosomal recessive. Skeletal dysplasias can affect the skeleton only, or be part of pleiotropic syndromes affecting multiple organs. Mutations of various genes within a molecular pathway can each produce similar phenotypes. Loss-of function (LoF) mutations completely disrupt the activities of their encoded proteins but hypomorphic mutations allowing reduced protein activities occur. Gain-of-function (GoF) mutations increase the activities of enzymes and receptors and produce different phenotypes than LoF mutations. Dominant-negative mutations adversely affect functions of wild-type proteins. Mutations can occur within the protein-coding region of the genome (exome), within introns, or between gene coding regions. Mutations include deletions, duplications, and inversions.

The 2019 edition of the ISDS Nosology and Classification of Skeletal Disorders database organizes mutant human skeletal phenotypes into 42 groups, based on clinical observations and known gene/phenotype relationships (8). A total of 461 disorders and 441 genes are provided, when all 10 genes listed within the Notes sections of the tables (Table 1) are included. Updated HGNU gene symbols for 11 genes (Table 2) are employed. Supplemental Table 1 provides an alphabetical list in spreadsheet format of all 441 genes, with information on heredity, gene function and mouse model status. Genetic disorders are not listed, as mutations in many genes result in multiple phenotypes. Inheritance patterns are 242 autosomal recessive, 135 autosomal dominant, 34 autosomal recessive or autosomal dominant depending upon the exact mutation in the gene, 21 X-linked and 11 non-inherited, somatic mutations. Three genes can have either germline or somatic mutations.

**Table 1:** Genes identified in 2019 Nosology notes section.

**Gene**	**Model status**	**Nosology notes comments**
AFF3 (LAF4 in notes)	Mouse model	Microdeletion on Chr 2
C2CD3	Mouse model	OFD phenotypes
COG1	No data	CDG type 2G
EED	Mouse	Weaver syndrome
LMBR1	Mouse model	Deletion affecting SHH ZRS
MACROH2A1 (H2AFY in notes)	Mouse model	Deletion—PITX1 ectopic activation
RASGRP2	Mouse	Osteopetrosis—leukocyte adhesion
SDC2	Mouse	Chr 8q22.1 duplication
SUZ12	Mouse model	Weaver syndrome
VANGL1 (STB2)	Mouse	Caudal regression—OMIM 600145

**Table 2:** Gene symbol nomenclature.

**Nosology gene symbol**	**HGNC gene symbol**
CIAS1	NLRP3
CDC45L	CDC45
PPGB	CTSA
DHPAT	GNPAT
EVC1	EVC
FAM58A	CCNQ
HSGNAT	HGSNAT
LEPRE1	P3H1
PCNT2	PCNT
WISP3	CCN6
ZAK	MAP3K20

*HGNC, Human genome organization gene nomenclature committee*.

*RMRP* encodes an RNA regulating DNA transcription, *RNU4ATAC* encodes an RNA that is a component of an enzyme complex, and *MIR140* is a microRNA. Proteins (and the 3 RNAs) function as enzymes (146, 33%), scaffold components (79, 18%), ligand/receptor signaling molecules (72, 16%), transcription factors (62, 14%), cilia components (36, 8%), matrix proteins (23, 5%), membrane transporters (19, 4%), and cohesionopathy proteins (4, 1%). These eight gene function categories are informative but arbitrary, and other categories can be envisioned. For example, 23 enzymes are involved in the synthesis, processing, and degradation of protein and glycosaminoglycan matrix components. Skeletal disorders include malfunctions of lysosomal function. Signaling genes can be assigned to BMP, FGF, WNT, and other pathways.

There are no orthologous mouse genes for human *ARSE* (arylsulfatase E) and *RNU4ATAC* (RNA, U4atac small nuclear, U12-dependent splicing). Supplemental Table 1 summarizes published data on the availability and fidelity of mouse models for the 439 human rare bone disease genes. Mutant mice with bone phenotypic data exist for 260 of the 439 genes (59%) with similar bone phenotypes observed for 249 (96%) genes. Supplemental Table 2 contains PubMed hyperlinks to publications for all 249 genes provided in Supplement Table 1 having mutant mouse bone phenotypes. These two supplemental tables should provide a major resource for the bone research community.

Mutant mouse bone data are inconsistent with human skeletal phenotypes for 11 genes (*Ccn6, Cyp2r1, Flna, Galns, Gna13, Lemd3, Manba, Mnx1, Nsd1, Plod1, Smarcal1*). There are no obvious explanations for or commonalities among these human-mouse phenotype inconsistencies. For 97 genes (22%) mutant mice have been generated and examined, but no skeletal data were reported. Mutant mice do not appear to have been examined for 82 genes (19%) and 36 (8%) of these genes belong to the understudied Ignorome/Dark Genome (18–20). Individual laboratories and/or consortia are encouraged to examine these genes, now known to contribute to poorly understood human rare bone diseases.

The number of bone nosology genes continues to increase as novel genes affecting skeletal metabolism are identified in human subjects. The genes described in this report form an arbitrary “snapshot” taken during August 2019 and will undoubtedly increase. Skeletal disorders for which mutant genes have not been identified include CDAGS syndrome (OMIM 603116), cherubism with gingival fibromatosis (OMIM 266270), chondrodysplasia punctata tibial-metacarpal type (OMIM 118651), dysplasia epiphysealis hemimelica (OMIM 127800), femur fibula ulna syndrome (OMIM 228200), hemifacial microsomia (OMIM 1642100, genochondromtosis (OMIM 1373600, Moreno–Nishimura–Schmidt syndrome (OMIM 608811), pachydermoperiostosis (OMIM 167100), and thoracolaryngopelvic dysplasia (OMIM 187760).

Formation of a normal skeleton involves BMP, FGF, and WNT signaling pathways and mutations in multiple genes within these pathways often produce skeletal dysplasias. Bone cells respond to parathyroid hormone, the active vitamin D metabolite calcitriol, and circulating FGF23 as part of the calcium-phosphate homeostatic system and disruptions in these hormones produce skeletal endocrinopathies. Skeletal disorders involving aggrecanopathies (13), channelopathies (21), ciliopathies (22, 23), cohesinopathies (24), lamiopathies (25), linkeropathies (26), protein-folding defects (27), ribosomopathies (28), spliceosomopathies (29), and transcription factors (30) show the importance of pathways not often thought to be involved in bone development.

## Skeletal Disorder Vignettes

This section briefly summarizes selected skeletal disorders resulting from various mutations, highlighting the wide range of transcription and translation events that can be disrupted.

- Mutations can be benign with healthy nutrition but produce disease when key nutrients are lacking. All humans have an inactivating mutation in GULO, encoding an enzyme involved in the synthesis of ascorbic acid, and develop scurvy without sufficient dietary intake of vitamin C. The ascorbate synthetic pathway, involving aldehyde and aldose reductases, was only fully characterized in 2010 (31). Ascorbic acid is a required cofactor for the hydroxylation of proline and lysine residues in collagen and disruption of the mouse gulonolactone oxidase gene results in spontaneous bone fractures (32). Similarly, human and mouse *HAAO* and *KYNU* genes are involved in the synthesis of the enzymatic cofactor NAD and inactivating mutations in these human and mouse genes can result in congenital malformations (33).
- X-linked human mutations comprise 6% of the total skeletal disorders. X-inactivation of one of the two X chromosomes in women by long non-coding RNA specific transcript *XIST* occurs, but about 20% of X chromosome genes escape this inactivation (34). *AMER1* and *PORCN* are X-linked genes that code for components of the WNT signaling pathway, with dominant mutations in women causing osteopathia striata with cranial sclerosis and focal dermal hypoplasia (including osteopathia striata), respectively. Due to developmental lethality male patients are extremely rare, but a few males having post-zygotic mosaic mutations have been identified (35, 36). *Amer1* mutations in mice disrupt bone architecture (37) and treating adult mice with inhibitors of the PORCN enzyme reduces bone mass (38).
- Somatic gene mutations in 11 genes (*AKT1, FLBN, GNAS, GREM1, HRAS, IDH1, IDH2, MAP2K1, NOTCH2, NRAS, PIK3CA*) arise in the developing zygote and are not transmitted genetically. Loeys-Dietz syndrome includes several skeletal dysplasias and can result from mutations in *SMAD2, SMAD3, TGFB2, TGFB3, TGFBR1*, or *TGFBR2* and 75% of affected subjects have somatic mutations (39). Melorheostotic, dense hyperostotic bone lesions are caused by somatic mosaic mutations in *KRAS* (40) and *MAP2K1* (41). *MAP2K1* mutations are thought to arise after the formation of dorso-ventral plane (42). *KRAS* and *MAP2K1* are not included among the 441 Nosology disorders. Mutations in *COL11A*1, *EZH2*, and *MET* can be either germline or somatic.
- Deleterious mutations can occur at multiple sites within genes. For example, there are 1053 *COL1A1* DNA variants in the *Osteogenesis Imperfecta Variant Database* as of September 2019 (https://oi.gene.le.ac.uk/home.php?select_db=COL1A1, accessed 13 December, 2019).
- Splicing mutations that disrupt normal exon transcription within the spliceosome are estimated to contribute to 15% of human genetic diseases (43, 44). Acrofacial and mandibulofacial dysostosis often involve spliceosome defects and mutations in *EFTUD2, EIF4A3*, and *SF3B4* genes each result in distinct craniofacial phenotypes. Splice site mutations in *AIFM1* (45), *SERPINF1* (46), and *TRAPPC2* (47) result in skeletal dysplasias.
- MicroRNAs are non-protein coding single-stranded RNAs (48) that regulate gene expression in bone and other tissues. Mouse studies show that microRNA-140 is involved in growth plate development (49, 50). A gain-of function mutation in microRNA-140 results in human skeletal dysplasia (51).
- Subjects with intragenic duplications of *IFT81* (tandem duplication of exons 9 and 10) and *MATN3* (tandem duplication of exons 2–5), detected by WGS, have skeletal dysplasias similar to subjects with LoF mutations in these genes (52).
- Autosomal-dominant syndactyly, synpolydactyly, and brachydactyly types D and E can result from dominant-negative mutations in the homeobox gene *HOXD13*. Duplications of the *HOXD* gene cluster locus produce mesomelic dysplasia with shortened limbs (53, 54). Similar *Hoxd* locus GoF alterations in *ulnaless* mutant mice, generated by X-irradiation, produce similar bone phenotypes (55, 56).
- ISDS nosology includes skeletal disorders resulting from disruptions of calcium-phosphate homeostasis, including various endocrinopathies. Regulation of calcium and phosphorus homeostasis involves *ALPL, CASR, DMP1, ENPP1, FAM20C, FGF23, GALANT3, HRAS, KL, NRAS* and *TRPV6* genes. Parathyroid hormone synthesis and action involve *CDC73, FAM111A, GCM and PTH1R*. Vitamin D synthesis and actions involve *CYP2R1, CYP27B1* and *VDR*. Normal Ca and P homeostasis occurs in humans (57) and mice (58) with deletions of the GC gene and thereby lacking the circulating vitamin D-binding protein (DBP) that binds serum 25-OH-D. Multiple neonatal bone fractures were observed due to maternal hypoparathyroidism and vitamin D deficiency (59).

## Heredity of Bone Mass Without Skeletal Dysplasia

Osteoporosis is a common skeletal disease in which reduced amounts of otherwise normal bone lead to fragility and fractures. Adult bone mass, even within the normal range, has a strong heredity influence (60, 61) and identifying the genes involved in bone mass accumulation during growth and loss during aging has received great interest within the context of the etiology and treatment of osteoporosis. GWAS studies over the past decade described an increasing number of genes affecting BMD, with 518 loci identified in the 2019 UK Biobank analysis (62). Juvenile osteoporosis, although not a true dysplasia as bone architecture is normal, usually has genetic causes (63, 64). There are healthy subjects with unexplained high bone mass (65, 66) and attempts are underway to identify the genes responsible. Recent discoveries of such genes include *LRP6* (67) and *SMAD9* (68).

## Mouse Models

*All models are wrong, but some are less imperfect than others, and many are useful* - George Box

Mouse models make important contributions to understanding and treating human diseases (69–72), including skeletal disorders (73, 74). Mutant mice that model human phenotypes also model successful drugs (75), help identify genes responsible for human genetic disorders and can provide insights for osteoporosis drug development (76). Bone mass and architecture vary in healthy humans and among laboratory mouse strains, with the most commonly studied C57BL/6 mouse strain an outlier having limb bones with high diameters and low cortical thickness (77–81).

The majority of mouse data summarized in this review involve individual investigator-initiated studies examining possible skeletal phenotypes in transgenic mice with specific alterations in genes chosen by the investigator. This approach, known as reverse genetics, utilizes the expertise of the laboratories involved.

In contrast, human studies involve forward genetics, with genes responsible for known skeletal phenotypes identified. Forward genetics is also employed in mouse studies, as genes responsible for spontaneous and mutagen-induced skeletal malformations are identified. The Jackson Laboratories (JAX), with a long history of studying mouse strains, recently employed WES to identify 14 genes having spontaneous mutations causing bone phenotypes (82, 83). Several laboratories employed N-ethyl-N-nitrosourea (ENU) in chemical mutagenesis campaigns to produce mouse lines having a wide-range of phenotypes. This approach yielded 41 genes having mutations causing bone phenotypes similar to the corresponding human skeletal disorders. These 41 genes with relevant citations are provided in Supplemental Table 3.

Two high-throughput mouse reverse genetics gene knockout phenotyping campaigns have been undertaken (84). The International Mouse Phenotyping Consortium (IMPC, www.mousephenotype.org) aims to characterize knockout mouse phenotypes for all 20,000 genes (74, 85). Lexicon Pharmaceuticals' Genome5000™ effort examining the druggable genome confirmed known bone phenotypes for 23 genes and identified 11 genes, including *Notum* (86), for which bone phenotypes were not previously characterized (87). Importantly, skeletal phenotypes were described for *Fam20c* (non-lethal Raine syndrome), *Lrrk1* (osteosclerotic metaphyseal dysplasia), *Pappa2* (short stature), *Sfrp4* (Pyle's disease), and *Slc10a7* (skeletal dysplasia) prior to knowledge of the human skeletal dysplasias when mutated in humans (84). For the 439 mouse genes discussed in this review, 149 genes have been examined by the IMPC, yielding 63 viable adult homozygous mouse mutants. Skeletal phenotypes (either body BMD or radiological dysmorphology) were observed for 28 genes. Results from the IMPC phenotyping campaign are summarized in Table 3.

**Table 3:** Summary of International Mouse Phenotyping Campaign (IMPC) models.

**Category**	**Number of genes**
Total mouse protein-coding genes^a^	437 (100%)
Genes not assigned for IMPC analyses	52 (12% of total)
Genes with ES cells generated, but no mice	183 (42% of total)
Mice generated without phenotyping	56 (13% of total)
Mouse phenotyping completed	149 (34% of total)
Embryonic and preweaning lethality	86 (58% of 149 phenotypes)
Subviable (Few surviving homozygous mice)^b^	7 (5% of 149 phenotypes)
Lack of bone data^c^	5 (3% of 149 phenotypes)
No observed bone phenotypes^d^	23 (15% of 149 phenotypes)
Bone phenotypes^e^	28 (19% of 149 phenotypes)

a *No mouse genes for human ARSE and RNU4ATAC; Mir140 and Rmrp are RNA-coding genes*.

b *Cant1, Chst14, Dnajc21, Dnmt3a, Dock6, Egot, and Zswim6*.

c *Skeleton not tested for Dmp1, Map3k20, Snx10 and Sulf1; no BMD data for Ltbp2*.

d *Bgn, Bhlha9, Cc2d2a, Cfap410, Cyp2r1, Gpc6, Haao, Ick, Idh1, Idh2, Knyu, Npr3, Orc4, Picb4, Ptdss1, Pycr1, Serpinf1, Smarcal1, Tctex1d2, Thpo, Tmem165, and Trappc2*.

e *Low BMD for Hdac8, Lpin2, Nek1, P3h1, Phex, Plod1, Pls3, Setd2, Sparc and Wnt10b; high BMD for Col1a2, Fuca1, Gnas, Hgsnat, Lrrk1, and Sgsh; skeletal dysmorphology for Col9a2, Creb3l1, Ctsk, Ift80, Mmp9, Plekhm1, Sh3bp2, Suz12; low BMD and dysmorphology for Cyp27b1; homozygous lethality with adult heterozygous dysmorphology for Pitx1 and Pthlp; and homozygous lethality with fetal dysmorphology and adult heterozygous dysmorphology for Nxn*.

Mouse models of human genetic disorders are employed to evaluate potentially beneficial skeletal actions of therapies approved for other disease indications. Teriparatide treatment increases bone mass in *Lrp5* KO mice mimicking humans with *osteoporosis pseudoglioma syndrome* from loss of function *LRP5* mutations (88, 89). Similarly, anti-sclerostin antibody treatment increases bone mass in mutant mouse models with low bone mass from gene disruptions (90) of *Col1a1* (91, 92), *Col1a2* (93, 94), *Crtap* (95), *Dmp1* (96), *Lrp5* (97), and *Zmpste24* (98). Mechanistic hypotheses can be tested, such as periostin treatment retarding skull suture fusion in heterozygous *Twist1* mice with craniosynostosis (99).

## Mouse Study Precautions

Several experimental pitfalls should be avoided when performing mouse studies (100).

- Knockout of individual genes can disrupt the functions of neighboring genes (101). Examples include the presence of orofacial defects resulting from a hypomorphic *Pax9* allele during knockout of the neighboring *Slc25a21* gene (102) and glycosaminoglycan accumulation resulting from reduced expression of the *Naglu* gene during knockout of the neighboring *Hsd17b1* gene (103).
- Transgenic Cre mouse lines are invaluable for conditionally activating or inactivating genes of interest. Several reporter genes are available for visualizing bone cells at different stages of development (104). But not all Cre lines are as specific as originally believed (105–107). Understanding these limitations is critical for experimental design and interpretation.
- Quantitative PCR methods are often not optimized and MIQE (Minimum Information for the publication of qPCR Experiments) guidelines have been established (108, 109). Selection of the appropriate reference gene(s) is important (110–112).
- Many antibodies suffer from a lack of specificity resulting from cross-reactivity to similar epitopes present on multiple proteins. Clifford Saper in 2005, as Editor-in-Chief of *The Journal of Comparative Neurology*, repeatedly received “… distressed communications from authors … to withdraw papers because an antibody against a novel marker was found to stain tissue in knockout animals …” (113). Excellent reviews (not cited here) provide guidelines for successful antibody validation and the purposeful joviality in their titles (“*Antibody Can Get It Right … Antibody Anarchy … Antibody Crimes … A Guide to the Perplexed … Garbage In, Garbage Out … Hitchhiker Antigens … Not for the Faint-Hearted … The Dark Side of the Immunohistochemical Moon … The Good, Bad, and Really Ugly*”) emphasizes the seriousness of the problem. Antibodies claimed to be specific for particular proteins should not react against tissues from KO mice missing the gene of interest and validation of antibody specificity using tissues from KO cells or mice is strongly encouraged.
- Established cell lines employed in conjunction with mouse studies can become contaminated and replaced by more robust, faster growing cells (114). Cell line authentication methods exist and should be employed (115, 116). MC3T3-E1 cell subclones vary as models of osteoblast biology (117).

## Large Animal and Zebrafish Models

Large animals can have advantages over rodents for understanding human genetic disease and drug development. Hypophosphatasia occurs in sheep (118) and dogs (119) having mutations in *ALPL*. Canine genetic skeletal disorders include mutations in *ADAMTSL2*—geleophysic dysplasia (120), *COL1A2*—osteogenesis imperfecta (121), *DVL2*—Robinow syndrome (122), *HES7*—spondylocostal dysostosis (123), and *SERPINH1*—osteogenesis imperfecta (124). Spontaneous mutations in chicken *KIAA0586* (125) *and LMBR1* (126) genes result in the expected bone phenotypes.

Zebrafish are increasing contributing to our knowledge of skeletal genomics (127, 128) and advantages over mouse models include acquiring data more rapidly. Zebrafish mutants have been described for several of the 441 genes in this review. One complication of zebrafish studies is that zebrafish underwent a teleost-specific whole genome duplication and have more than 26,000 protein-coding genes (129). There is a one-to-one relationship between 47% of human genes and a zebrafish ortholog. There are multiple zebrafish genes associated to a single human gene, and vice versa.

## Drug Development

Exciting advances are being made in developing drug treatments for patients with genetic skeletal disorders (130, 131) and mouse models invariably contribute to this progress. These advances are best reviewed by the laboratories involved, but three examples are illustrative. An antibody to NOTCH2 reverses osteopenia in a mouse model of Hajdu-Cheney syndrome (132). Cinacalcet corrects hypercalcemia in a mouse model of familial hypercalcemia type 2 (133). ENPP1 enzyme replacement therapy improves blood pressure and cardiovascular function in a mouse model of generalized arterial calcification of infancy (134).

Understanding genetic skeletal disorders provides key knowledge for developing osteoporosis therapies (76, 135). Disruptions in genes coding for proteins in the RANK—RANKL—osteoprotegerin signaling pathway involved in osteoclast generation cause human skeletal disorders. The RANKL neutralizing antibody denosumab is a successful osteoporosis therapy. The recently approved anabolic osteoporosis treatment romosozumab, a sclerostin neutralizing antibody, was developed with knowledge gained from subjects with osteosclerosis resulting from *SOST* gene mutations. Subjects with pinocytosis have mutations in the cathepsin K coding gene *CTSK*. Treatment with odanacatib, an inhibitor of cathepsin K in osteoclasts, reduced bone fractures in postmenopausal women but cardiovascular side effects precluded regulatory approval.

## Future Directions

Since many human disorders involve hypomorphic, gain-of-function, dominant-negative and intronic mutations, future studies will undoubtedly utilize CRISPR/Cas9 technology and other evolving techniques to examine transgenic mice having genes modified to exactly mimic variant human sequences (72, 136). RNA sequencing will increasingly be employed for diagnosis and mechanistic understanding of genetic diseases (137–141).

The IFMRS (International Federation of Musculoskeletal Research Societies), in collaboration with the Broad Institute, is establishing a Musculoskeletal Genomics Knowledge Portal (MGKP) to integrate, interpret and present human data linked to musculoskeletal traits and diseases (http://www.kp4cd.org/about/bone).

## Author Contributions

RB performed the literature search, data analyses, and prepared the manuscript. CO provided helpful suggestions and reviewed the manuscript.

### Conflict of Interest

The authors declare that the research was conducted in the absence of any commercial or financial relationships that could be construed as a potential conflict of interest.
