# Supplementary material for: High Fidelity of Mouse Models Mimicking Human Genetic Skeletal Disorders
Source: Front Endocrinol (Lausanne). 2020 Feb 4;10:934. doi: 10.3389/fendo.2019.00934 (PMC7010808; doi:10.3389/fendo.2019.00934)
Supplement: Supplementary file 3 [file Table_3.docx]

Supplemental Table 3: Mutant Mouse Skeletal Phenotypes from ENU Mutagenesis Campaigns

Mouse Gene Mouse Phenotypes Mouse Gene Publications

*Alpl* alkaline phosphatase deficiency Aigner, Hough, Sabrautzki

*Alx4* craniofacial, limb, digit, tail malformations MUTAGENETIX™ *goofy* mutant

*Arsb* high body BMD, craniofacial abnormalities Curtain (JAX Report)

*Car2* (*CA2*) vascular calcification Rajachar, Spicer

*Casr* ectopic calcification, hypercalcemia Hough, Sabrautzki

*Col1a1* osteogenesis imperfecta Chen, Lisse, Tabeta from MUTAGENETIX

*Col2a1* multiple skeletal dysplasias Cionni, Esapa, Furuichi

*Ednra* mandibulofacial dysostosis Sabrautzki

*Enpp1* low bone mass, ectopic calcification Babij, Li

*Fbn2* hindlimb syndactyly, muscle weakness Miller

*Fgfr1* craniofacial malformation Calvert

*Fgfr2* limb malformation, gonadal sex reversal Siggers

*Flnb* scoliosis, lordosis Dauphinee

*Galnt3* hyperphosphatemia, high bone mass Duncan, Esapa

*Gdf5* dominant-negative brachypodism, etc. Masuya

*Gja1* syndactyly, craniofacial malformation Flenniken

*Gnas* ectopic calcification Cheeseman

*Gnptab* craniofacial malformation, kyphosis Paton

*Ift140* craniofacial and digit abnormalities Miller

*Intu* polydactyly, endochondral ossification defects Chang

Kif7 preaxial polydactyly, embryonic lethality Liem

*Kl* (Klotho) ectopic calcification, osteopenia Esapa

*Lfng* limb, digit, tail malformations MUTAGENETIX™ *zigzag* mutant

*Lmbr1* preaxial polydactyly Masuya

*Lrp5* low bone mineral density Charette

*Ltbp3* limb, digit, tail malformations MUTAGENETIX™ *csp* mutant

*Mks1* limb patterning Weatherbee

*Mmp14* craniofacial defects MUTAGENETIX™ *cartoon* mutant

*Npr3* overgrowth, kyphosis Dauphinee, Esapa, MUTAGENETIX™ *eel* mutant

*Ostm1* mild osteopetrosis Bosman

*Phex* hypophosphatemic rickets Carpinelli, Owen, Sabrautzki, Xiong

*Shh* preaxial polydactyly Masuya

*Tcirg1* dominant-negative osteopetrosis Ochotny

*Tent5a* (*Fam46a*) multiple skeletal defects Diener

*Tnfs11* (Rankl) osteoporosis Douni

*Ttc21b* polydactyly, rib defects Tran, Strottman

*Twist1* polydactyly Blanc

*Wdr19* short limb, embryonic lethal Ashe

*Wdr35* polysyndactyly, embryonic lethal Mill

*Wnt7a* limb, digit, tail malformations MUTAGENETIX™ *gimpy* mutant

*Xylt1* disproportionate dwarfism Mis

The Mutagenetix™ database (<https://mutagenetix.utsouthwestern.edu>, accessed 23 January 2020) provides mouse ENU phenotype summary data for 360 genes (Wang et al. 2015; Wang et al., 2018).

Wang T, Zhan X, Bu CH, Lyon S, Pratt D, Hildebrand S, Choi JH, Zhang Z, Zeng M, Wang KW, Turer E, Chen Z, Zhang D, Yue T, Wang Y, Shi H, Wang J, Sun L, SoRelle J, McAlpine W, Hutchins N, Zhan X, Fina M, Gobert R, Quan J, Kreutzer M, Arnett S, Hawkins K, Leach A, Tate C, Daniel C, Reyna C, Prince L, Davis S, Purrington J, Bearden R, Weatherly J, White D, Russell J, Sun Q, Tang M, Li X, Scott L, Moresco EM, McInerney GM, Karlsson Hedestam GB, Xie Y, Beutler B. Real-time resolution of point mutations that cause phenovariance in mice. Proc Natl Acad Sci USA. 2015; 112:E440-E449.

Wang T, Bu CH, Hildebrand S, Jia G, Siggs OM, Lyon S, Pratt D, Scott L, Russell J, Ludwig S, Murray AR, Moresco EMY, Beutler B. Probability of phenotypically detectable protein damage by ENU-induced mutations in the Mutagenetix database. Nat Commun. 2018; 9:441.

Mouse ENU Mutant Gene Publications

Aigner B, Rathkolb B, Klaften M, Sedlmeier R, Klempt M, Wagner S, Michel D, Mayer U, Klopstock T, de Angelis MH, Wolf E. Generation of N-ethyl-N-nitrosourea-induced mouse mutants with deviations in plasma enzyme activities as novel organ-specific disease models. Exp Physiol. 2009; 94:412-421.

Ashe A, Butterfield NC, Town L, Courtney AD, Cooper AN, Ferguson C, Barry R, Olsson F, Liem KF Jr, Parton RG, Wainwright BJ, Anderson KV, Whitelaw E, Wicking C. Mutations in mouse Ift144 model the craniofacial, limb and rib defects in skeletal ciliopathies. Hum Mol Genet. 2012; 21:1808-1823.

Babij P, Roudier M, Graves T, Han CY, Chhoa M, Li CM, Juan T, Morony S, Grisanti M, Li X, Yu L, Dwyer D, Lloyd DJ, Bass MB, Richards WG, Ebeling C, Amato J, Carlson G. New variants in the Enpp1 and Ptpn6 genes cause low BMD, crystal-related arthropathy, and vascular calcification. J Bone Miner Res. 2009; 24:1552-1564.

Blanc I, Bach A, Lallemand Y, Perrin-Schmitt F, Guénet JL, Robert B. A new mouse limb mutation identifies a Twist allele that requires interacting loci on chromosome 4 for its phenotypic expression. Mamm Genome. 2003; 14:797-804.

Bosman EA, Estabel J, Ismail O, Podrini C, White JK, Steel KP. Omi, a recessive mutation on chromosome 10, is a novel allele of Ostm1. Mamm Genome. 2013; 24:44-53.

Carpinelli MR, Wicks IP, Sims NA, O'Donnell K, Hanzinikolas K, Burt R, Foote SJ, Bahlo M, Alexander WS, Hilton DJ. An ethyl-nitrosourea-induced point mutation in phex causes exon skipping, x-linked hypophosphatemia, and rickets. Am J Pathol. 2002; 161:1925-1933.

Calvert JA, Dedos SG, Hawker K, Fleming M, Lewis MA, Steel KP. A missense mutation in Fgfr1 causes ear and skull defects in hush puppy mice. Mamm Genome. 2011; 22:290-305.

Chang R, Petersen JR, Niswander LA, Liu A. A hypomorphic allele reveals an important role of inturned in mouse skeletal development. Dev Dyn. 2015; 244:736-747.

Charette JR, Earp SE, Bell BA, Ackert-Bicknell CL, Godfrey DA, Rao S, Anand-Apte B, Nishina PM, Peachey NS. A mutagenesis-derived Lrp5 mouse mutant with abnormal retinal vasculature and low bone mineral density. Mol Vis. 2017; 23:140-148.

Cheeseman MT, Vowell K, Hough TA, Jones L, Pathak P, Tyrer HE, Kelly M, Cox R, Warren MV, Peters J. A mouse model for osseous heteroplasia. PLoS One. 2012; 7:e51835

Chen F, Guo R, Itoh S, Moreno L, Rosenthal E, Zappitelli T, Zirngibl RA, Flenniken A, Cole W, Grynpas M, Osborne LR, Vogel W, Adamson L, Rossant J, Aubin JE. First mouse model for combined osteogenesis imperfecta and Ehlers-Danlos syndrome. J Bone Miner Res. 2014; 29:1412-1423.

Cionni M, Menke C, Stottmann RW. The mouse MC13 mutant is a novel ENU mutation in collagen type II, alpha 1. PLoS One. 2014; 9:e116104.

Curtain MM, Donahue LR. A mutation in the Arsb gene; a mouse model that resembles mucopolysaccha-ridosis type VI (MPS VI). <http://www.informatics.jax.org/downloads/Reference_texts/J149960.pdf>

Dauphinee SM, Eva MM, Yuki KE, Herman M, Vidal SM, Malo D. Characterization of two ENU-induced mutations affecting mouse skeletal morphology. G3 (Bethesda). 2013; 3:1753-1758.

Diener S, Bayer S, Sabrautzki S, Wieland T, Mentrup B, Przemeck GK, Rathkolb B, Graf E, Hans W, Fuchs H, Horsch M, Schwarzmayr T, Wolf E, Klopocki E, Jakob F, Strom TM, Hrabě de Angelis M, Lorenz-Depiereux B. Exome sequencing identifies a nonsense mutation in Fam46a associated with bone abnormalities in a new mouse model for skeletal dysplasia. Mamm Genome. 2016; 27:111-121.

Douni E, Rinotas V, Makrinou E, Zwerina J, Penninger JM, Eliopoulos E, Schett G, Kollias G. A RANKL G278R mutation causing osteopetrosis identifies a functional amino acid essential for trimer assembly in RANKL and TNF. Hum Mol Genet. 2012; 21:784-798.

Duncan EL, Danoy P, Kemp JP, Leo PJ, McCloskey E, Nicholson GC, Eastell R, Prince RL, Eisman JA, Jones G, Sambrook PN, Reid IR, Dennison EM, Wark J, Richards JB, Uitterlinden AG, Spector TD, Esapa C, Cox RD, Brown SD, Thakker RV, Addison KA, Bradbury LA, Center JR, Cooper C, Cremin C, Estrada K, Felsenberg D, Glüer CC, Hadler J, Henry MJ, Hofman A, Kotowicz MA, Makovey J, Nguyen SC, Nguyen TV, Pasco JA, Pryce K, Reid DM, Rivadeneira F, Roux C, Stefansson K, Styrkarsdottir U, Thorleifsson G, Tichawangana R, Evans DM, Brown MA. Genome-wide association study using extreme truncate selection identifies novel genes affecting bone mineral density and fracture risk. PLoS Genet. 2011; 7:e1001372.

Esapa CT, Head RA, Jeyabalan J, Evans H, Hough TA, Cheeseman MT, McNally EG, Carr AJ, Thomas GP, Brown MA, Croucher PI, Brown SD, Cox RD, Thakker RV. A mouse with an N-Ethyl-N-nitrosourea (ENU) Induced Trp589Arg Galnt3 mutation represents a model for hyperphosphataemic familial tumoural calcinosis. PLoS One. 2012; 7:e43205.

Esapa CT, Hough TA, Testori S, Head RA, Crane EA, Chan CP, Evans H, Bassett JH, Tylzanowski P, McNally EG, Carr AJ, Boyde A, Howell PG, Clark A, Williams GR, Brown MA, Croucher PI, Nesbit MA, Brown SD, Cox RD, Cheeseman MT, Thakker RV. A mouse model for spondyloepiphyseal dysplasia congenita with secondary osteoarthritis due to a Col2a1 mutation. J Bone Miner Res. 2012; 27:413-428.

Esapa CT, Hannan FM, Babinsky VN, Potter P, Thomas GP, Croucher PI, Brown MA, Brown SD, Cox RD, Thakker RV. N-ethyl-N-Nitrosourea (ENU) induced mutations within the klotho gene lead to ectopic calcification and reduced lifespan in mouse models. PLoS One. 2015; 10:e0122650.

Esapa CT, Piret SE, Nesbit MA, Loh NY, Thomas G, Croucher PI, Brown MA, Brown SD, Cox RD, Thakker RV. Mice with an N-Ethyl-N-Nitrosourea (ENU) induced Tyr209Asn mutation in natriuretic peptide receptor 3 (NPR3) provide a model for kyphosis associated with activation of the MAPK signaling pathway. PLoS One. 2016; 11:e0167916.

Flenniken AM, Osborne LR, Anderson N, Ciliberti N, Fleming C, Gittens JE, Gong XQ, Kelsey LB, Lounsbury C, Moreno L, Nieman BJ, Peterson K, Qu D, Roscoe W, Shao Q, Tong D, Veitch GI, Voronina I, Vukobradovic I, Wood GA, Zhu Y, Zirngibl RA, Aubin JE, Bai D, Bruneau BG, Grynpas M, Henderson JE, Henkelman RM, McKerlie C, Sled JG, Stanford WL, Laird DW, Kidder GM, Adamson SL, Rossant J. A Gja1 missense mutation in a mouse model of oculodentodigital dysplasia. Development. 2005; 132:4375-4386.

Furuichi T, Masuya H, Murakami T, Nishida K, Nishimura G, Suzuki T, Imaizumi K, Kudo T, Ohkawa K, Wakana S, Ikegawa S. ENU-induced missense mutation in the C-propeptide coding region of Col2a1 creates a mouse model of platyspondylic lethal skeletal dysplasia, Torrance type. Mamm Genome. 2011 Jun;22(5-6):318-328.

Hough TA, Bogani D, Cheeseman MT, Favor J, Nesbit MA, Thakker RV, Lyon MF. Activating calcium-sensing receptor mutation in the mouse is associated with cataracts and ectopic calcification. Proc Natl Acad Sci USA. 2004; 101:13566-713571.

Hough TA, Polewski M, Johnson K, Cheeseman M, Nolan PM, Vizor L, Rastan S, Boyde A, Pritzker K, Hunter AJ, Fisher EM, Terkeltaub R, Brown SD. Novel mouse model of autosomal semidominant adult hypophosphatasia has a splice site mutation in the tissue nonspecific alkaline phosphatase gene Akp2.

J Bone Miner Res. 2007; 22:1397-1407.

Li Q, Guo H, Chou DW, Berndt A, Sundberg JP, Uitto J. Mutant Enpp1asj mice as a model for generalized arterial calcification of infancy. Dis Model Mech. 2013; 6:1227-1235.

Liem KF Jr, He M, Ocbina PJ, Anderson KV. Mouse Kif7/Costal2 is a cilia-associated protein that regulates Sonic hedgehog signaling. Proc Natl Acad Sci USA. 2009; 106:13377-133782.

Lisse TS, Thiele F, Fuchs H, Hans W, Przemeck GK, Abe K, Rathkolb B, Quintanilla-Martinez L, Hoelzlwimmer G, Helfrich M, Wolf E, Ralston SH, Hrabé de Angelis M. ER stress-mediated apoptosis in a new mouse model of osteogenesis imperfecta. PLoS Genet. 2008; 4:e7.

Masuya H, Nishida K, Furuichi T, Toki H, Nishimura G, Kawabata H, Yokoyama H, Yoshida A, Tominaga S, Nagano J, Shimizu A, Wakana S, Gondo Y, Noda T, Shiroishi T, Ikegawa S. A novel dominant-negative mutation in Gdf5 generated by ENU mutagenesis impairs joint formation and causes osteoarthritis in mice. Hum Mol Genet. 2007; 16:2366-2375.

Masuya H, Sezutsu H, Sakuraba Y, Sagai T, Hosoya M, Kaneda H, Miura I, Kobayashi K, Sumiyama K, Shimizu A, Nagano J, Yokoyama H, Kaneko S, Sakurai N, Okagaki Y, Noda T, Wakana S, Gondo Y, Shiroishi T. A series of ENU-induced single-base substitutions in a long-range cis-element altering Sonic hedgehog expression in the developing mouse limb bud. Genomics. 2007; 89:207-214.

Mill P, Lockhart PJ, Fitzpatrick E, Mountford HS, Hall EA, Reijns MA, Keighren M, Bahlo M, Bromhead CJ, Budd P, Aftimos S, Delatycki MB, Savarirayan R, Jackson IJ, Amor DJ. Human and mouse mutations in WDR35 cause short-rib polydactyly syndromes due to abnormal ciliogenesis. Am J Hum Genet. 2011; 88:508-515.

Miller G, Neilan M, Chia R, Gheryani N, Holt N, Charbit A, Wells S, Tucci V, Lalanne Z, Denny P, Fisher EM, Cheeseman M, Askew GN, Dear TN. ENU mutagenesis reveals a novel phenotype of reduced limb strength in mice lacking fibrillin 2. PLoS One. 2010; 5:e9137.

Miller KA, Ah-Cann CJ, Welfare MF, Tan TY, Pope K, Caruana G, Freckmann ML, Savarirayan R, Bertram JF, Dobbie MS, Bateman JF, Farlie PG. Cauli: a mouse strain with an Ift140 mutation that results in a skeletal ciliopathy modelling Jeune syndrome. PLoS Genet. 2013; 9:e1003746.

Mis EK, Liem KF Jr, Kong Y, Schwartz NB, Domowicz M, Weatherbee SD. Forward genetics defines Xylt1 as a key, conserved regulator of early chondrocyte maturation and skeletal length. Dev Biol. 2014; 385:67-82.

Ochotny N, Flenniken AM, Owen C, Voronov I, Zirngibl RA, Osborne LR, Henderson JE, Adamson SL, Rossant J, Manolson MF, Aubin JE. The V-ATPase a3 subunit mutation R740S is dominant negative and results in osteopetrosis in mice. J Bone Miner Res. 2011; 26:1484-1493.

Owen C, Chen F, Flenniken AM, Osborne LR, Ichikawa S, Adamson SL, Rossant J, Aubin JE. A novel Phex mutation in a new mouse model of hypophosphatemic rickets. J Cell Biochem. 2012; 113:2432-2441.

Paton L, Bitoun E, Kenyon J, Priestman DA, Oliver PL, Edwards B, Platt FM, Davies KE. A novel mouse model of a patient mucolipidosis II mutation recapitulates disease pathology. J Biol Chem. 2014; 289:26709-26721.

Rajachar RM, Tung E, Truong AQ, Look A, Giachelli CM. Role of carbonic anhydrase II in ectopic calcification. Cardiovasc Pathol. 2009; 18:77-82.

Sabrautzki S, Rubio-Aliaga I, Hans W, Fuchs H, Rathkolb B, Calzada-Wack J, Cohrs CM, Klaften M, Seedorf H, Eck S, Benet-Pagès A, Favor J, Esposito I, Strom TM, Wolf E, Lorenz-Depiereux B, Hrabě de Angelis M. New mouse models for metabolic bone diseases generated by genome-wide ENU mutagenesis. Mamm Genome. 2012; 23:416-430.

Sabrautzki S, Sandholzer MA, Lorenz-Depiereux B, Brommage R, Przemeck G, Vargas Panesso IL, Vernaleken A, Garrett L, Baron K, Yildirim AO, Rozman J, Rathkolb B, Gau C, Hans W, Hoelter SM, Marschall S, Stoeger C, Becker L, Fuchs H, Gailus-Durner V, Klingenspor M, Klopstock T, Lengger C, Stefanie L, Wolf E, Strom TM, Wurst W, de Angelis MH. Viable Ednra Y129F mice feature human mandibulofacial dysostosis with alopecia (MFDA) syndrome due to the homologue mutation. Mamm Genome. 2016; 27:587-598.

Siggers P, Carré GA, Bogani D, Warr N, Wells S, Hilton H, Esapa C, Hajihosseini MK, Greenfield A. A novel mouse Fgfr2 mutant, hobbyhorse (hob), exhibits complete XY gonadal sex reversal. PLoS One. 2014; 9:e100447.

Spicer SS, Lewis SE, Tashian RE, Schulte BA. Mice carrying a CAR-2 null allele lack carbonic anhydrase II immunohistochemically and show vascular calcification. Am J Pathol. 1989; 134:947-954.

Stottmann RW, Tran PV, Turbe-Doan A, Beier DR. Ttc21b is required to restrict sonic hedgehog activity in the developing mouse forebrain. Dev Biol. 2009; 335:166-178.

Tabeta K, Du X, Arimatsu K, Yokoji M, Takahashi N, Amizuka N, Hasegawa T, Crozat K, Maekawa T, Miyauchi S, Matsuda Y, Ida T, Kaku M, Hoebe K, Ohno K, Yoshie H, Yamazaki K, Moresco EMY, Beutler B. An ENU-induced splice site mutation of mouse Col1a1 causing recessive osteogenesis imperfecta and revealing a novel splicing rescue. Sci Rep. 2017; 7:11717.

Tran PV, Haycraft CJ, Besschetnova TY, Turbe-Doan A, Stottmann RW, Herron BJ, Chesebro AL, Qiu H, Scherz PJ, Shah JV, Yoder BK, Beier DR. THM1 negatively modulates mouse sonic hedgehog signal transduction and affects retrograde intraflagellar transport in cilia. Nat Genet. 2008; 40:403-410.

Weatherbee SD, Niswander LA, Anderson KV. A mouse model for Meckel syndrome reveals Mks1 is required for ciliogenesis and Hedgehog signaling. Hum Mol Genet. 2009; 18:4565-4575.

Xiong X, Qi X, Ge X, Gu P, Zhao J, Zhao Q, Gao X. A novel Phex mutation with defective glycosylation causes hypophosphatemia and rickets in mice. J Biomed Sci. 2008; 15:47-59.
